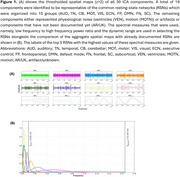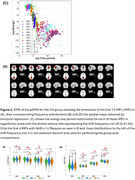# Energy characterization of the AD continuum using the Empirical Mode Decomposition approach for resting state fMRI in ADNI

**DOI:** 10.1002/alz70856_107126

**Published:** 2026-01-08

**Authors:** Pavithran Pattiam Giriprakash, Filippo Cieri, Xiaowei Zhuang, Zhengshi Yang, Jessica ZK Caldwell, Dietmar Cordes

**Affiliations:** ^1^ Cleveland Clinic Lou Ruvo Center for Brain Health, Las Vegas, NV, USA; ^2^ Cleveland Clinic, Las Vegas, NV, USA; ^3^ University of Nevada Las Vegas, Las Vegas, NV, USA; ^4^ Nevada Exploratory Alzheimer's Disease Research Center, Las Vegas, NV, USA

## Abstract

**Background:**

Time‐frequency analysis of resting‐state fMRI (rs‐fMRI) is essential for uncovering intrinsic frequency and amplitude characteristics. Mean energy and frequency profiles of different resting‐state networks (RSNs) can provide fundamental information about brain activity and its impairment in aging, and characterize stage‐specific alterations across the Alzheimer's disease (AD) continuum: cognitively normal (CN), mild cognitive impairment (MCI), and AD.

**Method:**

Using the ADNI database (adni.loni.usc.edu), a total of 297 fMRI sessions from 150 participants (all positive for amyloid PET) were included in this study. We determined RSNs using standard group ICA software. Then, using Empirical Mode Decomposition (EMD), all RSN time series were decomposed into intrinsic mode functions (IMFs). Only the first 4 IMFs that spanned a frequency range above 0.01 Hz were used for the characterization of the RSNs. We estimated energy and frequency measures to characterize our 3 groups.

**Result:**

With respect to the mean energy profiles, we found significantly reduced energy in the diseased groups (MCI and AD) in IMF2 and IMF3 of many RSNs with reference to the controls. For certain RSNs, specifically, frontoparietal; visual; temporal; the IMF4 showed the opposite trend (with large effect size, Cohen's d>0.8) with the AD group having increased mean energy compared to the other two groups. In terms of the frequency profiles a similar increase in the mean frequency was observed for IMF2, IMF3 and IMF4 in the AD and MCI patients with respect to the controls.

**Conclusion:**

We found that MCI and AD participants showed reduced energy and increased frequency in many RSNs. In particular, the DMN network (DMN1) showed the largest group difference in energy and frequency for IMF2 and IMF3. This is consistent with the fact that low‐frequency power (<0.1 Hz) is more related with cognitive function, while high‐frequency power (>0.1 Hz) is more associated with physiological activity. From a clinical perspective the decreased energy shown by the AD patients for many RSNs highlights the potential of fMRI in capturing differences between diagnostic groups. Specifically, these variables could act as potential biomarkers that models the characteristic changes in different brain networks along the AD continuum.